# Mechanical loading and orthobiologic therapies in the treatment of post-traumatic osteoarthritis (PTOA): a comprehensive review

**DOI:** 10.3389/fbioe.2024.1401207

**Published:** 2024-06-24

**Authors:** Mahammad Gardashli, Max Baron, Charles Huang, Lee D. Kaplan, Zhipeng Meng, Dimitrios Kouroupis, Thomas M. Best

**Affiliations:** ^1^ Department of Education, Miller School of Medicine, University of Miami, Miami, FL, United States; ^2^ Department of Biomedical Engineering, University of Miami, Miami, FL, United States; ^3^ Department of Orthopedics, UHealth Sports Medicine Institute, Miller School of Medicine, University of Miami, Miami, FL, United States; ^4^ Department of Molecular and Cellular Pharmacology and Sylvester Comprehensive Cancer Center, University of Miami Miller School of Medicine, Miami, FL, United States; ^5^ Diabetes Research Institute and Cell Transplant Center, Miller School of Medicine, University of Miami, Miami, FL, United States

**Keywords:** post-traumatic osteoarthritis, orthobiologics, mechanical loading, exercise, physical activity

## Abstract

The importance of mechanical loading and its relationship to orthobiologic therapies in the treatment of post-traumatic osteoarthritis (PTOA) is beginning to receive attention. This review explores the current efficacy of orthobiologic interventions, notably platelet-rich plasma (PRP), bone marrow aspirate (BMA), and mesenchymal stem/stromal cells (MSCs), in combating PTOA drawing from a comprehensive review of both preclinical animal models and human clinical studies. This review suggests why mechanical joint loading, such as running, might improve outcomes in PTOA management in conjunction with orthiobiologic administration. Accumulating evidence underscores the influence of mechanical loading on chondrocyte behavior and its pivotal role in PTOA pathogenesis. Dynamic loading has been identified as a key factor for optimal articular cartilage (AC) health and function, offering the potential to slow down or even reverse PTOA progression. We hypothesize that integrating the activation of mechanotransduction pathways with orthobiologic treatment strategies may hold a key to mitigating or even preventing PTOA development. Specific loading patterns incorporating exercise and physical activity for optimal joint health remain to be defined, particularly in the clinical setting following joint trauma.

## 1 Post-traumatic osteoarthritis–pathophysiology

Post-traumatic osteoarthritis (PTOA) is a severe condition that frequently follows traumatic injuries to the joints, such as fractures or damage to the surrounding soft tissues (like cartilage, ligaments, tendons, and meniscus), as well as the periarticular muscles. It represents a distinct subset of osteoarthritis (OA) triggered by such injuries, resulting in the onset of cartilage loss, narrowing of the joint space, and the formation of osteophytes, all hallmarks of OA progression. Approximately 27 million Americans experience symptomatic OA, with PTOA accounting for 10%–12% of diagnoses. What’s particularly concerning is that individuals with isolated anterior cruciate ligament (ACL) tears have a PTOA prevalence ranging from 0% to 39%, but when combined with meniscus tears, the prevalence rises significantly to 21%–100% (Thomas et al., 2017). In terms of socioeconomic burden, treating lower extremity PTOA incurred a cost of $11.79 billion in 2005, with direct costs surpassing $3 billion (Thomas et al., 2017). With an aging population and projections indicating the highest percentage of geriatric patients in the near future, PTOA is poised to become an increasingly pressing public health concern, necessitating proactive measures for management and intervention.

The breadth of research on PTOA is somewhat limited, hindering our understanding of its pathogenesis, and effective treatment strategies to mitigate its onset and progression. Interestingly, there is growing recognition that PTOA is a distinct OA phenotype. Such phenotyping will arguably lead to more specific treatment strategies that may differ from non-traumatic OA. In fact, in a pioneering study involving genetically modified mice, the molecular pathophysiology of PTOA and age-related non-traumatic OA was shown to differ between the two conditions (Lane et al., 2011; Little and Hunter, 2013). Recently, a clinical investigation reported different knee movements, muscle activation patterns, and variable gait-related risk factors for disease progression between PTOA and non-traumatic OA (Robbins et al., 2021). Importantly, inflammation plays a key role in inducing knee PTOA disease progression. Multiple studies highlight the increase in inflammatory biomarkers both systemically and locally that influence the progression of PTOA (Herman and Gobbi, 2023). Within the joint’s milieu, cellular communication between the adjacent synovium and infrapatellar fat pad (IFP) has been shown to contribute to fibrosis and inflammation mediated by cytokines like TNF-a and IFN-g, leading to OA development (Macchi et al., 2018; Greif et al., 2020).

**FIGURE 1:**
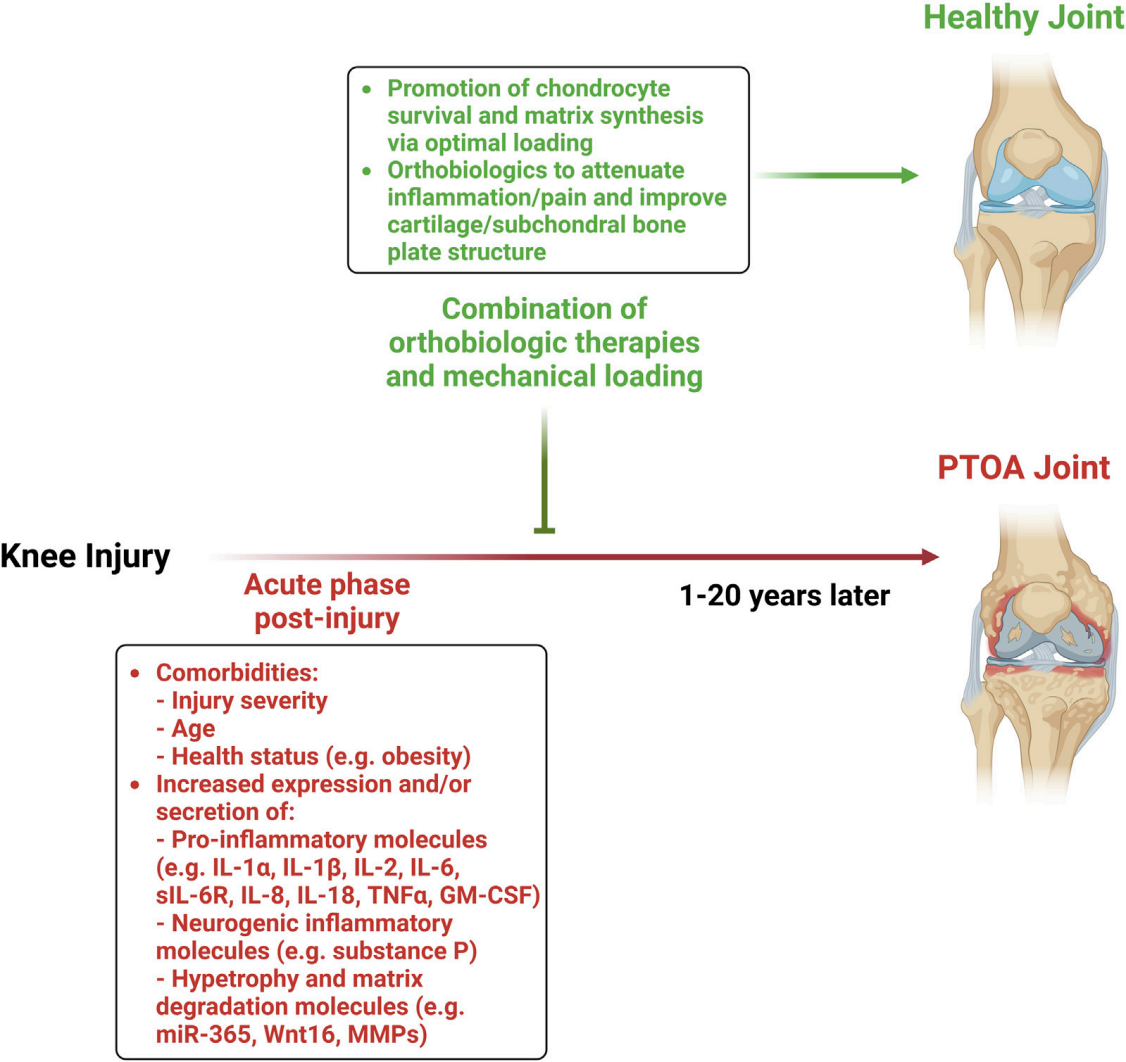
Combination of orthobiologic therapies and mechanical loading to mitigate the development of PTOA.

Whether these pathophysiological differences observed in pre-clinical and clinical models translate into different treatment strategies for humans remains unknown and warrants further investigation. One promising approach is the early post-injury application of orthobiologics—biologically derived materials aimed at modulating regeneration and repair—combined with appropriately dosed mechanical loading. This strategy has the potential to reduce inflammation and prevent cartilage degradation cascades, potentially mitigating the development of PTOA (Figure 1).

### 1.1 Preclinical and clinical studies on PTOA

Preclinical models refer to experimental studies conducted on animals, *in vitro*, and *ex vivo* systems to investigate the mechanisms and potential treatments for PTOA before they are applied to humans. Clinical models, on the other hand, involve research conducted on human subjects, including observational studies and clinical trials.

Recent preclinical studies have provided valuable insight into the pathophysiological mechanisms of PTOA (Table 1). Clinical and radiological including articular cartilage (AC) degeneration, synovitis, synovial fluid accumulation, subchondral bone exposure and sclerosis, ectopic bone formation, and osteophyte formation have been identified to assess PTOA incidence and progression (Croen et al., 2023; Kuyinu et al., 2016). Although, PTOA and atraumatic OA symptomatology are typically similar, factors contributing to their development may be different. Specifically, PTOA stems from injury to the AC, injury to the subchondral bone, misalignment of the joint surface, or joint instability resulting from sudden trauma such as joint fracture or ligament injury (Kramer et al., 2011). A non-invasive mouse model of compressive joint loading demonstrated key features mimicking human PTOA including cartilage degeneration, synovitis, and subchondral bone changes (Bhatti et al., 2022). All of these findings correlated with development of pian in humans at risk for PTOA (Khella et al., 2021; Croen et al., 2023). It has been also shown in preclinical models that ACL surgical transection (ACLs), noninvasive ACL rupture (ACLc), and destabilization of the medial meniscus (DMM) can lead to PTOA with DMM resulting in milder progression and ACLs showing more rapid advancement (Croen et al., 2023; Maerz et al., 2015). Croen et al. found that the ACL injury groups showed higher expression of pain markers and genes associated with muscle atrophy, with pain-related gait behavior appearing earlier in the ACL rupture groups than in the DMM condition. High-resolution imaging and histology indicated differing bone microstructure changes between ACLs and DMM models, suggesting distinct mechanical loading environments (Croen et al., 2023).

**TABLE 1:** Summary of animal studies investigating the effects of different running intensities on the development of PTOA.

References	Animal model	Therapeutic groups	Control groups	Intensity of running	Follow up	Histopathology assessment	Effect of running on PTOA development
Tsai et al. (2019)	Medial meniscal transection rat model (*n* = 21)	- Hindlimb immobilization group - Treadmill running group	- No intervention group - Sham group	- Low - Moderate - High	8 weeks	- All three MMT groups showed full-thickness lesions and/or increased cartilage volume compared to the sham group - The no intervention and treadmill-running groups had greater osteophyte formation than the hindlimb immobilization group	- Low or moderate-speed running did not accelerate PTOA - High speed running increase PTOA
Zhou et al. (2021)	- Twenty-week-old C57BL/6 mouse model (*n* = 30) - High-intensity treadmill running for 4 weeks to create PTOA	- Treadmill model group (M) - Treadmill model + rehabilitation training group (M + R) - Treadmill model + convalescent group (M + C)	- Model control group (MC) - Rehabilitation control group (RC)	- Moderate - High	4 weeks	- Mankin’s and OARSI scores M > MC - Mankin’s and OARSI scores M + C > M + R > RC	Moderate-intensity running increased the expression of lncRNA H19 in cartilage and potentially relieved motion-induced PTOA. High intensity running decreased lncRNA H19 expression in cartilage, suggesting an increased risk of knee injury and accelerated PTOA.

A recent systematic review and meta-analysis of randomized controlled trials and cohort studies identified risk factors for PTOA following traumatic knee injury in patients aged greater than 30 at time of injury. Significant risk factors included injury severity, patient age, obesity, and the presence of meniscal, ACL, or chondral injuries (Whittaker et al., 2022). Injury severity was found to be the strongest predictor of PTOA, particularly knee dislocation. Patients with meniscal injuries or a combination of ACL and meniscal injuries also had an increased risk of developing PTOA. Interestingly, the study revealed no significant difference in PTOA risk between patients who ACL reconstruction and patients who underwent meniscal repair (Whittaker et al., 2022). Additionally, the study’s meta-analysis revealed that male sex at ACL tear does not increase the chances of developing structural OA (Whittaker et al., 2022). In a long-term population-based study skeletally immature patients who suffered a first-time patellar dislocation at a mean age of 30 were investigated for their propensity to develop PTOA. Results demonstrated a high rate of recurrent dislocation and a subsequent development of PTOA in 20% of patients (Cuzzolin et al., 2021). The male to female ratio in this systematic review and meta-analysis was 2:3. During this post-traumatic knee injury period the complex local inflammatory response is associated with a 4 to 10-fold increased risk of PTOA (Nieboer et al., 2023). Specifically, IL-6 and CCL4 pro-inflammatory cytokines were consistently shown to be elevated in the synovial fluid within the first 6 weeks in patients age stratified to 45 years or younger post-injury, suggesting the involvement of early inflammatory markers in PTOA development (Nieboer et al., 2023). Intra-articular fractures have also been implicated as a reason for PTOA development. In a pioneering study, Volpin et al. examined 31 patients 6–22 years after intra-articular fracture of the knee and found that 23% of patients develop PTOA regardless of surgical or conservative treatment (Volpin et al., 1990). The pathophysiology of intra-articular fractures inducing PTOA has been recently described, showing that acute mechanical damage can lead to chondrocyte apoptosis. Specifically, chondrocyte injury induces sGAG releasement from the matrix of human cartilage explants within 96 h after injury indicating cell death and apoptosis (McKinley et al., 2010; D’Lima et al., 2001).

### 1.2 Mechanisms of gene regulation in PTOA

In a review by Hodgkinson et al., it was observed that within a PTOA mouse model, Wnt16 protein and mRNA were upregulated (Hodgkinson et al., 2022; Nalesso et al., 2017). Interestingly, Hodgkinson et al. also described a study which revealed that Wnt16 deficient had more severe OA than wildtype mice suggesting a potential homeostatic function of Wnt16 in the progression of PTOA (Hodgkinson et al., 2022; Nalesso et al., 2017). Additionally, miR-365, which governs cell hypertrophy through NF-κB and HDAC4 mechanisms, is elevated by cyclical loading in human and rat chondrocytes and is also overexpressed in cartilage from patients with age-related or PTOA (Hodgkinson et al., 2022; Yang et al., 2016).

Importantly, in a porcine model of ACL disruption and repair, inflammatory markers (including interleukin IL-1α, IL-1β, IL-2, IL-6, IL-8, IL-18, TNFα, and GM-CSF) were elevated within 3 days following surgery suggesting their presence may play a crucial role in the pathogenesis of PTOA (Zhao et al., 2021; Han et al., 2018). A more recent study harvested osteochondral explants from human ankle talocrural joints and demonstrated that inflammatory cytokines TNFα, IL-6, and sIL-6R effectively established an environment to investigate early PTOA-like disease initiation (Szapary et al., 2023). These cytokines reduced cell viability, intensified sulfated glycosaminoglycan (sGAG) loss, and heightened nitric oxide release (Szapary et al., 2023). Their findings underscore a strong correlation between sGAG content and biomechanical properties in human talus cartilage, particularly impacting the equilibrium modulus and dynamic stiffness (Szapary et al., 2023). Additionally, a recent study examined the role of clock gene Bmal1 in PTOA-related cartilage degeneration. Decreased Bmal1 expression was observed in osteoarthritic and aging cartilage, whereas Bmal1 deletion in cartilage increased mTORC1 activity, leading to chondrocyte apoptosis and cartilage degradation. These results suggest a significant role of Bmal1 in PTOA progression through the regulation of mTORC1 and its activities in AC and chondrocytes (Qian et al., 2023). Substance P, derived from nociceptive nerve fibers, functions in transmitting pain signals and has been shown to regulate local inflammation. It plays an additional role involved in the vasodilation of peripheral vessels, aiding the movement of monocytes from circulation to neighboring tissues (Greif et al., 2020). Moreover, Substance P has been identified as an inducer for a unique M2 phenotype. M2 macrophages play a major role in anti-inflammatory responses throughout the body. Substance P induced M2SP, a phagocytic macrophage involved in tissue repair and distinct from the established M2a and M2c subtypes, thereby contributing to specialized macrophage differentiation involved in the inflammatory cascade (Lim et al., 2017).

There are many changes in gene expression associated with the pathogenesis of PTOA. Different studies have used techniques to classify the genetic changes within the joint as PTOA progresses. In a previous study, an ACL transection and medial meniscectomy rat model was used to study the sequential changes in gene expression during early PTOA progression. Transcriptomic analysis of AC revealed 849 and 223 differentially expressed genes at 2 weeks and 4 weeks after surgery, respectively. Interestingly, 22 novel genes were identified. Among those Gnai1, Sema4d, Plxnb1, and Srgap2 are part of axon guidance pathway, which had never been reported to be involved in PTOA progression, were identified as key factors in cartilage degeneration (Liu et al., 2021). Another study, examined the transcriptional profile of AC in Yucatan minipigs following ACL transection. Cartilage collected at 1- and 4-week post-surgery showed significant microscopic degeneration compared to controls, with similar gene expression patterns across untreated, reconstruction, and ligament repair groups post ACL transection. Specifically, *MMP1, COCH, POSTN, CYTL1, PTGFR, ADAMTS4, SERPINB7, CR2,* and *INHBA* showed differential expression at both 1 and 4 weeks, pointing to the activation of multiple proteases (including MMP1 and ADAMTS4) in early PTOA stages (Sieker et al., 2018). Importantly, a recent study investigated how aging impacts PTOA development. Structural and molecular changes were studied in a non-invasive tibial compression injury model of 10- and 62-week-old mice. At 6-weeks post-injury, 62-week-old mice showed more cartilage degeneration and osteophyte formation compared to 10-week-old mice. While both groups had similar transcriptional responses to injury, the 62-week-old mice had a stronger inflammatory response, whereas the younger mice had higher expression of genes related to cartilage and bone metabolism. This difference in gene expression may account for the varying severity of PTOA between the age groups (Sebastian et al., 2020). Conclusively, previous studies provide us not only with the gene expression changes related to the pathogenesis of PTOA but also with information regarding the time course of gene expression and how aging affects it.

## 2 Orthobiologic therapies and treatment of PTOA

As inflammation and fibrosis play key roles in PTOA progression, it may prove valuable to develop innovative therapies that can attenuate the unchecked immune cascades and ideally promote tissue regeneration. This dual focus of modulating both inflammation and fibrosis may be essential for effective interventions, particularly when exploring the regenerative capabilities of mesenchymal stem/stromal cells (MSCs) and platelet-rich plasma (PRP) in the treatment of PTOA. These treatments, often derived from autologous sources, tend to exhibit fewer adverse effects compared to traditional therapies of corticosteroid injections and immunosuppressive agents, and are less invasive compared to other orthobiologic therapies such as chondrocyte transplantation and implantation. Consequently, MSCs and PRP offer considerable promise for safe and effective clinical application in managing PTOA (Hermann et al., 2018; Thoene et al., 2023).

### 2.1 Platelet-rich plasma (PRP)

Platelet-rich plasma (PRP) is an autologous platelet solution concentrated above physiological levels. Historically, these supraphysiologic levels were achieved through repeated centrifugations. The regenerative capabilities of platelet alpha granules are the basis for PRP therapy. *In vivo*, during wound healing platelets undergo degranulation and release growth factors (VEGF, IGF-1) and chemokines that coordinate tissue regeneration (Wu et al., 2016; Boswell et al., 2012). Also, at the site of wound healing, leukocytes play a pivotal role in removing damaged cells and tissue repair. However, their presence in PRP therapy has been implicated in proinflammatory states and a marked reduction in healing. Several *in vitro* studies have shown that leukocyte-rich PRP (LR-PRP) induces cell death and suggests using leukocyte-poor PRP (LP-PRP) for regenerative therapies in human (Braun et al., 2014; Assirelli et al., 2015). On the other hand, many studies claim LR-PRP therapies are of value, considering that leukocytes, such as neutrophils and macrophages, have potent anti-inflammatory properties in tissue regeneration (Lana et al., 2019). These studies focus on leukocyte inflammatory mediators as an essential component for wound healing claiming that depending on the method of preparation, blood derivative composition, and disease setting, LR-PRP produces better outcomes (Lana et al., 2019; Filardo et al., 2021). However, the composition of PRP, including platelet and growth factor concentration, can be standardized based on the method of platelet activation, centrifugal metrics, the volume of blood collected, and the type of anticoagulants used such as sodium citrate (SC) verses ethylenediaminetetraacetic acid (EDTA) offering hope for protocols that could be compared in future studies (Kikuchi et al., 2019; Muthu et al., 2022; Carvalho et al., 2024).

### 2.2 PRP application in PTOA preclinical models

Given the relatively recent understanding of PTOA as a distinct phenotype of OA within the orthopedic field, it’s not unexpected that only two animal models have addressed PRP’s efficacy in PTOA treatment (Table 2). The first, in 2017, investigated the therapeutic potential of repeated injections involving three distinct modalities in mice: a solution derived from hyaluronan (HYADD-4G) administered in isolation, PRP alone, and a combined approach that incorporated both the hyaluronan derivative and PRP. These three groups were compared against a control group of mice that received injections of phosphate buffer saline (PBS) only. PTOA was induced following one bout of 60 cycles of 9N axial tibial compression for 0.34 s per cycle. The first intraarticular injection was performed the same day (day 0) following the tibial compression injury and on Days 28 and 42 followed by animal sacrifice at day 56. The study found that no treatment group (HA-derivative alone, PRP alone, or in combination) had a protective response against cartilage proteoglycan loss or chondrocyte loss at day 56. Furthermore, there was no significant deviation when compared to the control PBS-administered mice across various parameters, including synovitis, ectopic calcification reversal, trabecular bone volume fraction, and subchondral bone thickness (Duan et al., 2017). Another study focused on investigating the effects of LP-PRP and LR-PRP compared to PBS and sham controls in a PTOA mouse model. Specifically, authors repeated articular injections of LP-PRP, LR-PRP, and PBS at day 2, 7, and 28 post destabilization of the medial meniscus (DMM) surgery and analyzed OA pathogenesis through histology and computed tomography at 14 weeks. Overall, results showed no therapeutic effects of either PRP group in reduction in synovitis when compared to either control group. However, CT analysis highlighted a significant difference with increased cartilage thickness and greater cartilage surface area within the LP-PRP group compared to the PBS group. Additionally, the analgesic potential of PRP therapy in alleviating OA-induced pain, employing a hot plate nociceptive assay at weeks 5, 9, and 13 has been investigated. The LR-PRP group exhibited a reduction in thermal hyperalgesia when compared to the PBS group (Jayaram et al., 2020). Collectively, both studies showed modest/low therapeutic effects of PRP on PTOA, but these effects may be influenced by PRP composition such as leukocyte concentration (Colombini et al., 2019). However, as previously discussed, prematurely dismissing PRP therapy solely due to the lack of universally standardized methods seems unwarranted at this time, especially considering the minimal risks associated with PRP therapies and their potential clinical benefits. Therefore, the absence of a universally endorsed preparation method underscores the evident need for refining PRP extraction techniques. (Fadadu et al., 2019; Xiong et al., 2023).

**TABLE 2:** Summary of animal studies investigating the effects of PRP on PTOA.

References	Animal model	Therapeutic group	Control group	Follow up post -injection	Histopathology assessment	Outcomes
Duan et al. (2017)	Axial tibial loading 10-week-old C57BL/6J mouse model (*n* = 86; 6N and 9N loading)	−8 mg/mL HYADD-4G group −15 mg/mL HYADD-4G group - PRP (contaning 46.87 ng/mL TGF-β1 and 2.68 ng/mL PDGF AB) group −8 mg/mL HYADD-4G + PRP group	- PBS group	56 days	- OA score (based on OARSI proteoglycan scoring) after loading showed no significant effect of any treatment modality on proteoglycan content - Synovitis score in each group indicated that synovitis progressed with time but no significant differences were found in the same loading force groups at the same time point	No significant differences in synovitis, reversing ectopic calcification, trabecular bone volume fraction, subchondral bone thickness, cartilage proteoglycan loss or protecting/reversing chondrocyte loss comparing any experimental group to PBS controls
Jayaram et al. (2020)	Destabilization of medial meniscus wild-type FVB/N mouse model (n = 43)	- Leukocyte poor-PRP (LP-PRP) - Leukocyte rich-PRP (LR-PRP)	- PBS group - Sham group	5, 9, 13 and 14 weeks	Both LP-PRP and LR-PRP treatments had higher OARSI and synovitis scores compared to sham, and neither substantially improved scores compared to PBS group	LP-PRP had significantly higher cartilage thickness and surface area compared to PBS while LR-PRP had higher analgesic effects against OA induced pain

### 2.3 PRP application in clinical PTOA

For patients with knee OA, PRP therapy has become an increasingly popular treatment option. A previous study explored the effects of PRP therapy on symptomatic post-traumatic knee OA. The study enrolled 122 patients [60 with PTOA and 62 with knee trauma (KT)] and injected three doses of autologous PRP into 62 patients (*n* = 32 PTOA, *n* = 30 with KT) on top of standard treatment using pharmacologic (NSAIDS) and physical therapy interventions (exercises, complex physiotherapy).

All patients received pharmacologic and physical therapies; however, the specific exercises and loading conditions accompanying physical therapy were not documented. There results showed PRP treatment resulted in improved patient quality of life, determined by KOOS score, at early and late follow-ups compared to pharmacologic and physical therapy alone in both PTOA and KT patients (Havryliuk and Khimion, 2019). Interestingly, PRP therapy is not only explored as a treatment but also as a possible intervention. In an ongoing clinical trial 80 patients have been enrolled and effects of LP-PRP injections immediately following anterior cruciate ligament (ACL) reconstruction are being investigated. Participants are being followed for 3 years to determine if PRP treatment reduces the risk or progression of OA after a traumatic knee injury (Momoi et al., 2022).

### 2.4 Bone marrow aspirate

Bone marrow aspirate (BMA) is a promising orthobiologic therapy due to its rich composition of clotting factors, various hematopoietic cell types, and valuable stem cells, rendering its secretome a potential therapeutic agent for healing. The posterior iliac crest has gained popularity as a favored source of BMA due to its abundant cell count and easy accessibility (Lana et al., 2021). The primary applications of BMA involve enhancing cartilage regeneration. This is achieved through hypothesized clot formation, stabilizing a scaffold that facilitates the differentiation of cellular products toward chondrogenic lineage by BMA stem cells. Concentrating the BMA to boost mononuclear cells and elevate stem cell numbers beyond baseline levels has shown increased therapeutic efficacy (Hernigou et al., 2006). This concentrated solution is reintroduced via intra-articular injection or surgical implantation, often using hyaluronic acid or collagen derivatives as supportive scaffolds (Buda et al., 2010; Gobbi et al., 2014). Extensive research has evaluated BMA’s potential as a treatment for osteoarthritis (Kim et al., 2020), demonstrating notable improvements in defect filling and tissue regeneration in animal models of OA (Buda et al., 2010; Gigante et al., 2012; Gobbi et al., 2014). In clinical studies, BMA has exhibited varying yet generally promising results in reducing pain scores among OA patients (Kim et al., 2014; Shapiro et al., 2017). Yet, there have been no animal or human studies utilizing BMA for OA treatment that categorized patients according to the etiology of trauma. Hence, understanding BMA’s effectiveness in addressing PTOA invites further research.

### 2.5 Properties of mesenchymal stem/stromal cells (MSCs)

The prominence of adult stem cell technologies has significantly increased over the past 30 years. Mesenchymal stem/stromal cells (MSCs) are multipotent stromal cells found in various tissues of the body, responsible for restoring connective tissue such as AC (Gonzalez and Bernad, 2012). Additionally, MSCs possess a unique secretome that facilitates paracrine signaling, resulting in immunomodulatory effects in surrounding tissues. As a result, MSCs have emerged as potential therapeutic candidates for conditions such as OA and AC degeneration, with clinical evidence demonstrating positive outcomes following their administration (Colombini et al., 2019; Lopa et al., 2019). A significant portion of these effects have been attributed to MSC-secreted exosomes, which have shown the ability to modulate the inflammatory secretory profiles of macrophages (Kouroupis et al., 2022; Kouroupis et al., 2023).

Importantly, several studies have examined the safety of MSC therapies and found their use to be generally safe without significant risk of cellular transformation or tumorigenicity (Wang et al., 2021; von Bahr et al., 2012; Centeno et al., 2016; Hernigou et al., 2013). Clinically, MSC therapies can be applied either in autologous or allogeneic settings. Autologous MSCs are most commonly obtained from a patient’s bone marrow (BM-MSCs) or adipose (AD-MSCs) tissue and then fresh or upon *in vitro* expansion re-administered to the same patient for treatment (Centeno and Pastoriza, 2020). However, logistical challenges arise due to variations in the quantity and quality of MSCs among patients, and the consistent need for surgical collection for administration. As an alternative, allogeneic MSCs most commonly obtained from perinatal tissues (umbilical cord tissue, umbilical cord blood, and placenta), can be manufactured on a larger scale, resulting in consistent cellular products that are economically feasible and readily available (Kot et al., 2019; Vega et al., 2015).

### 2.6 MSC applications in PTOA preclinical models

While there is little clinical research exploring MSCs in the treatment of PTOA, several studies have investigated their therapeutic potential in various preclinical models (Table 3). In a caprine goat study, researchers injected BM-MSCs following complete excision of the medial meniscus and resection of the ACL recapitulating one of the more common models of human PTOA involving athletes. MSC therapy provided significant improvements in cartilage structure, including thickening of the subchondral bone plate (Murphy et al., 2003). In a similar study, authors injected BM-MSCs after unilateral transection of the ACL in a rabbit model and reported significantly better histopathological scores and less severe radiological signs of OA compared to controls (medium without cells) (Singh et al., 2014). In another study, chondrogenic-induced BM-MSCs were compared to standard BM-MSCs in a sheep PTOA model. Both groups showed reduced OA progression compared to the control group injected with basal media without cells; however, the chondrogenic-induced BM-MSCs displayed more promise in meniscus repair (Al Faqeh et al., 2012).

**TABLE 3:** Summary of animal studies investigating the effects of various types of MSCs on PTOA.

References	Animal model	Therapeutic group	Control group	Follow up post -injection	Histopathology assessment	Outcomes
Murphy et al. (2003)	ACL resection/medial meniscus excision goat model (*n* = 24)	-Single injection of GFP transduced caprine BM-MSCs in sodium hyaluronan (2 × 10^6^ cells/mL, 5 mL total) group	- Single injection of sodium hyaluronan group	6 and 20 weeks	Histologic scores for all parameters (articular cartilage structure, reduction of articular cartilage matrix staining, the presence of osteophytes, subchondral bone plate thickening) were closer to normal in the middle medial condyle of BM-MSCs treated group	BM-MSC treated group had a significant effect on subchondral bone plate thickening and AC maintenance structure compared to hyaluronan only controls
Singh et al. (2014)	ACL unilateral transection rabbit model (*n* = 20)	- Single injection of rabbit BM-MSCs (1 × 10^6^/ml, 1 mL total) group	- Single injection of 1 mL of medium without cells group	4 and 8 weeks	- The study group had significantly lower Mankin and Kellgren scores than the controls at both 4 and 8 weeks	The therapeutic group had significant improvements in histology and radiology associated with OA compared to medium only controls
Al Faqeh et al. (2012)	ACL excision sheep model (*n* = 16)	- Single injection of sheep chondrogenic induced BM-MSCs (2 × 10^6^/ml, 5 mL total) group	- Single injection of sheep non- induced BM-MSCs (2 × 10^6^/ml, 5 mL total) group	6 weeks	No significant difference was detected between the two groups in ICRS scoring	An advantage was reported in using chondrocyte induced BM-MSCs over BM-MSCs controls on meniscus regeneration, but both the control and therapeutic groups resulted in reduction of OA progression
Toghraie et al. (2011)	ACL transection rabbit model (n = 28 total)	- Singe injection of rabbit AD-MSCs (1 × 10^6^/ml, 1 mL total) group	- Sham group - Single injection of 1 mL of medium without cells group	12, 16, 20 weeks	- Mankin score showed no obvious difference between AD-MSCs treated and control groups at 16 weeks - Mankin score was significantly higher at control group compared to AD-MSCs treated group at 20 weeks	Animals receiving AD-MSCs treatment had a lower degree of cartilage loss, osteophyte formation, subchondral sclerosis, and overall qualitative improvements in the cartilage compared to both sham and media injection groups
Desando et al. (2013)	ACL bilateral transection rabbit model (n = 72)	- Single injection of rabbit AD-MSCS (2×10^6^ cells, 1 mL total) in 4% rabbit serum albumin (RSA) group - Single injection of rabbit AD-MSCs (6×10^6^, 1 mL total) in 4% rabbit serum albumin (RSA) group	- Single injection of 1 mL of 4 %RSA - Sham group - No intervention group	16 and 24 weeks	- AD-MSCS administration determined a decrease of Laverty’s scores compared to 4% RSA at both experimental times	AD-MSCs promoted anabolic processes for tissue formation and resulted in a reduced expression of TNF-α and MMP-1 compared to media only injection controls - Both AD-MSC groups demonstrated healing potential for the AC, but the lower concentration was more effective at repair
Wakayama et al. (2022)	Medial meniscus unilateral transection C57BL/6 mouse model (*n* = 20)	- Injection of human AD-MSCs (2 × 10^4^/μL, 6 μL total) group at 14, 28 and 42 days	- Injection of PBS group at 14, 28 and 42 days	42 days after third injection	OARSI score was significantly lower in the AD-MSCs group compared with control at the medial and lateral femorotibial joint	- AD-MSC injection to suppress AC loss - AD-MSC released anti-inflammatory cytokines into the media suggesting a paracrine mechanism of action
Lamers et al. (2020)	Medial meniscus unilateral release sheep model (n = 23)	- Single injection of 500 μL hyaluronic acid followed by human UC-MSCs (1×10^7^ in 1 mL) group	- Control group receiving a single injection of saline - Sham group receiving the cell therapy	13 weeks	The UC-MSCs group had improved OARSI total score and subscores compared to the group with OA	- UC-MSC injections caused a transient increase in CD4^+^ cell infiltrates with a large portion of them expressing CD25, the latter being a subset that may be composed of regulatory T cells - iNOS expression of intimal lining macrophages was evident but reduced in the UC-MSCs therapy group suggesting macrophage phenotype transformation - Cell therapy induced chemotaxis of CD4^+^ cells to the joint but these cells were not associated with pathological changes
Horie et al. (2009)	Medial meniscus excision rat model (*n* = 23)	- Single injection of rat Luc/LacZ^+^ S-MSCs (5×10^6^ cells in 50 μL PBS) group - Single injection of rat BM-MSCs (5×10^6^ cells in 50 μL PBS) group	- Single injection of PBS group	2, 4, 8 and 12 weeks	None documented	- S-MSC group showed improved meniscal regeneration compared to PBS group - S-MSCs adhered to the lesion, differentiated into meniscal cells directly, and promoted meniscal regeneration without mobilization to distant organs
Hatsushika et al. (2013)	Medial meniscus resection rabbit model (*n* = 16)	- Single injection of rabbit S-MSCs (1×10^7^ cells in 100 μL PBS) group	- Single injection of PBS group	4, 12, 16, and 24 weeks	Modified Pauli’s, and OARSI scores were significantly better in the S-MSCs group than in the control group	- Defect or thinning of the AC with sclerosis of the subchondral bone was observed in the control group, contrarily articular cartilage and subchondral bone were better preserved in the S-MSCs group - S-MSCs adhered around the meniscal defect, and promoted meniscal regeneration
Hatsushika et al. (2014)	Medial meniscus resection pig model (*n* = 7)	- Injection of pig S-MSCs (5×10^7^ cells in 1 mL of PBS) group at 0, 2 and 4 weeks	- Injection of PBS group at 0, 2 and 4 weeks	4, 8, and 12 weeks after third injection	Modified Pauli’s, ICRS, and OARSI scores were significantly better in the S-MSCs group than in the control group	There was significant meniscus regeneration and AC preservation in the S-MSC group compared to the control knee after histological and radiological examination

Similarly, multiple studies using AD-MSCs found improvement in cartilage integrity, structure, and thickness following ACL transection (Toghraie et al., 2011; Toghraie et al., 2012; Desando et al., 2013). Furthermore, a recent study showed that AD-MSC administration slowed PTOA progression in a medial meniscus destabilization mouse model attributed to the anti-inflammatory properties of MSCs (Wakayama et al., 2022). In another study, umbilical cord-derived MSC (UC-MSC) administration caused a transient increase in CD4 T cells with a large portion expressing CD25 regulatory T cell marker in synovial fluid. However, the effects of UC-MSCs on cartilage integrity were not evaluated. These results demonstrate the immunomodulatory effects of MSCs through the induction of regulatory T cells in the knee joint microenvironment (Lamers et al., 2020).

To further improve MSC therapeutic effect in the knee joint, many groups have used synovium-derived MSCs (S-MSCs) due to similarities in target tissue and ease of tissue access. Two separate groups have studied the regeneration capacity of S-MSCs in rat and rabbit animal models, respectively. Both groups showed that autologously injected S-MSCs improved meniscal regeneration compared to PBS injected animal groups after partial meniscectomy (Horie et al., 2009; Hatsushika et al., 2013; Hatsushika et al., 2014). While the S-MSC studies focus on meniscal regeneration rather than slowing OA progression, meniscal trauma often precedes PTOA and therefore, S-MSC therapy can potentially regenerate lost meniscal tissue and potentially delay OA onset (Englund et al., 2012).

### 2.7 MSC application in PTOA patients

As MSC therapy for PTOA is an evolving line of investigation, few clinical studies have demonstrated efficacy and safety. A pioneering study compared autologous BM-MSCs to autologous chondrocyte implantation (ACI) harvested from osteochondral fragments of the knee (Giannini et al., 2010). The MSC treatment group received an arthroscopic injection of 2 mL of autologously harvested bone marrow concentrate from the posterior iliac crest combined with 1 mL of platelet-rich fibrin gel prior to administration. Eighty-one patients with unilateral post-traumatic lesions were enrolled with 12- and 36-month follow-up. BM-MSC arthroscopic administration into the ankle joint relatively safe, with one patient having an antibiotic-resolved infection. There was no difference in pain and function (AOFAS score) when comparing arthroscopic BM-MSCs (*n* = 25) to both arthroscopic ACI (*n* = 46) and open-field ACI (*n* = 10). Compared to baseline, each group had an AOFAS score improvement at both follow-up time points.

A separate study investigated the safety, tolerability, and efficacy of intra-articular injection of allogenic BM-MSCs with hyaluronic acid (*n* = 11) compared to hyaluronic acid alone (*n* = 6). This phase Ib/IIa study focused on patients who underwent ACL reconstruction within 6 months of a traumatic injury and observed for changes in clinical symptoms and structural deterioration. Notably, no treatment-related serious adverse event was reported with 24-month follow up. The BM-MSCs with HA group demonstrated significant improvements in KOOS pain and symptom scores, and SF-36 bodily pain scoring at 18 and 24 months. Interestingly, MRI and x-ray imaging showed that the BM-MSC group had a significant reduction in medial and lateral tibiofemoral joint space narrowing and less tibial bone expansion than hyaluronic acid alone (Wang et al., 2017).

### 2.8 Differences in outcomes in animals vs. humans for orthobiologic therapies

In a recent review, de Girolamo et al. (2023) elucidated the disparity between the efficacy of various orthobiologic therapies in animal models of PTOA and their outcomes in human clinical trials (de Girolamo et al., 2023). Blood-derived products, adipose tissue-derived products, and bone marrow-derived products have demonstrated differing degrees of clinical effectiveness and potential disease-modifying effects (DMoE) in preclinical research models. Blood-derived products exhibited a clinical effect of over 80% and a DMoE of around 68%. Adipose tissue-derived products showed slightly lower clinical effects at 78% but higher DMoE at approximately 90%. Bone marrow-derived products demonstrated a clinical effect similar to blood-derived products at around 79%, with a DMoE of approximately 85% (de Girolamo et al., 2023). Despite these promising results in animal studies, translating such efficacy to human PTOA has proven inconsistent. This inconsistency is likely not due to differences in loading conditions. It has been shown that animal models are able to create a similar mechanical environment to the knee joint in a clinical model. A potential reason for the disparities between clinical and pre-clinical investigations is that clinical studies often do not investigate the same outcomes that are extensively evaluated in pre-clinical animal models. Most clinical studies prioritize examining clinical outcomes, often dedicating fewer resources to thoroughly evaluate DMoE’s such as the use of histological and immunohistochemical analysis (de Girolamo et al., 2023). This discrepancy underscores the need for further investigation into the mechanisms underlying orthobiologic efficacy and its translation to human clinical settings.

It is important to note that while both human and animal research can provide valuable insight into the role of mechanical loading on PTOA development, there are physiological differences in load bearing and anatomy between species, which is a major limitation when using animal models. Certain small animal models have been specifically designed and employed to investigate PTOA (Little and Hunter, 2013; Rios et al., 2022; Blaker et al., 2017). Small animals are favored due to their simplicity and cost-effectiveness. Doubts persist regarding their predictive accuracy, primarily because novel therapeutic effectiveness demonstrated in preclinical studies often fails to translate to human clinical trials (Rios et al., 2022; Malfait and Tortorella, 2019). Larger animal models offer advantages due to their anatomical resemblance to humans, thus enhancing their potential for translational research (Rios et al., 2022; Gregory et al., 2012).

## 3 Mechanotherapies and PTOA

The utilization of orthobiologics (such as MSCs and/or PRP) attempting to mitigate development of PTOA is on the rise, yet their efficacy in prevention of PTOA progression and treatment remains inconclusive. The variability in treatment outcomes could be attributed, in part, to the diverse loading patterns experienced by affected joints post-therapy. Therefore, investigating the impact of mechanical loading on joint health and identifying optimal loading patterns in conjunction with different biological therapies emerges as a potential approach refining treatment strategies.

Mechanotherapy, a field exploring how mechanical loading influences musculoskeletal health, offers valuable insights in this regard. For instance, a study by Zheng et al. demonstrated that subjecting mice to dynamic loading (1 N at 5 Hz for 5 min/d) enhanced their resistance to cartilage destruction and reduced Osteoarthritis Research Society International (OARSI) scores following medial meniscus removal, mimicking the progression of osteoarthritis (Zheng et al., 2019). While the focus of the study is not exclusively on post-traumatic OA compared to general osteoarthritis, the induction of medial meniscus removal aligns with a common PTOA-inducing condition. Both PTOA and tendinopathies are highly inflammatory processes leading to tissue architecture destruction and subsequent clinical symptoms. Wu et al. studied PTOA progression in a murine animal model comparing treatment with systemic bisphosphonate injection versus mechanical loading (force of 1.8 N for 5 min at a frequency of 4 Hz) and found less cartilage destruction in the mechanical loading group compared to bisphosphonate injections (Wu et al., 2023). Similar OA models found improvements in subchondral bone and osteoclast inhibition utilizing various loading regimens (Li et al., 2016; Zheng et al., 2020).

On this basis, our research (Hazbun et al., 2021) has investigated the effects of tibial axial loading (TAL) on porcine knee AC following a controlled traumatic injury. Using a custom TAL device, synovial fluid inflammatory biomarkers IL-1B and TNF-a along with AC degradation were measured before and after the loading intervention with a magnitude of 1/4 body weight at a frequency of 1 Hz for 30 min. Following acute knee joint injury, there was a significant decrease in pro-inflammatory cytokines IL-1B and TNF-a with TAL when compared to the injured porcine group without TAL (Hazbun et al., 2021). Our findings suggest that this dose of mechanical loading may downregulate knee joint inflammation and mitigate AC breakdown following a single impact injury, potentially reducing the risk for development of PTOA. Another preclinical study investigated the impact of knee joint loading on chondrocyte mechano-vulnerability and PTOA severity induced by ACL injury in mice (Willick and Hansen, 2010). A dose-response effect of mechanical loading in maintaining chondrocyte homeostasis was demonstrated (Kotelsky et al., 2022). Specifically, unilateral ACL injured mice balance their joint-loading between injured and uninjured hind limbs resulting in reduced joint-loading during gait. This altered gait resulted in improved chondrocyte viability and function while reducing inflammation and slowing PTOA progression. Conversely, bilateral ACL injury mice experienced higher joint-loading in both injured limbs thus resulting in increased chondrocyte death, increased inflammation, and greater PTOA severity. Interestingly, a recent study investigated the impact of timing on the effectiveness of mechanical loading and intra-peritoneal alendronate (ALN) injection on OA progression. Mice with OA induced by ACL transection were subjectee to either early (1–3 weeks) or late (5–7 weeks) axial compressive dynamic loading or ALN injection (Wu et al., 2023). Early loading and ALN treatments both had positive effects by reducing cartilage destruction and inflammation protection subchondral bone deterioration (Wu et al., 2023). However, delayed loading acclerated AC degeneration, suggesting the potential importance of relative load reduction in advanced OA (Wu et al., 2023).

There have been recent advancements in the field of mechanobiology, emphasizing the importance of continuous passive motion in PTOA patients. Evidence supports the significance of tribological loading in cartilage and chondrogenic cell physiology, highlighting the necessity for mimicking complex mechanical stimuli *in vitro* to better understand physiological responses that could result in improved translational outcomes (Oshida, 2007; Ladner and Stoddart, 2024). Multiaxial load bioreactors have emerged as valuable tools, offering advantages over simplistic loading devices by providing more physiologically relevant mechanical stimulation (Hannoun et al., 2021). These bioreactors enable the investigation of tribological loading effects on cartilage and chondrogenic cells and the maintenance of tissue lubrication and potential for expression of regenerative molecules (Ladner and Stoddart, 2024). Additionally, the development of innovative “tribo-bioreactors” facilitates *in-situ* monitoring of mechanical stress transmission at the cellular level, providing real-time evaluation of physical parameters during cell culture (Hannoun et al., 2021). Such advancements hold promise for optimizing loading protocols essential for cartilage tissue engineering and regenerative rehabilitation strategies, potentially leading to improved patient outcomes in PTOA management (Ladner and Stoddart, 2024).

## 4 Optimal loading patterns for articular cartilage health

Healthy AC facilitates smooth joint motions and the absorption of forces up to six times an individual’s body weight during activities such as running and jumping. It is a specialized connective tissue with a thickness of 2–4 mm, providing a frictionless surface for efficient movement. AC is an avascular, aneural structure consisting primarily of chondrocytes and extracellular matrix (ECM). The ECM contains a high-water content, collagen networks, sulfated glycosaminoglycans (sGAG), and proteoglycans (Sophia Fox et al., 2009; Eckstein et al., 2006). Chondrocytes synthesize collagen fibers and proteoglycans, maintaining ECM composition whereas proteoglycans reduce compression forces by forming a gel-like matrix through cross-linking with hyaluronan (HA). Collagen fibers as a part of the matrix contribute to joint tensile properties. The distribution of these components varies across different zones of the cartilage matrix, with the superficial zone having a higher water content while the deepest layer is richer in proteoglycans (Arokoski et al., 2000; Carballo et al., 2017). The various elements discussed above within AC contribute to the understanding of tissue biomechanics during the loading of joints.

Mechanical loading has a significant effect on chondrocyte function via a similar mechanism in which loading activated chondrocyte cell pathways contribute to cartilage homeostasis. Chondrocytes sense their physical environment via a vast array of mechanosensitive receptors that activate a network of downstream signaling pathways to regulate cell processes. Excessive loads can lead to cell death and inflammation. Static loading, at certain levels, can inhibit matrix formation. Dynamic loading, on the other hand, is considered the most favorable as it promotes ECM synthesis *ex vivo* (Sanchez-Adams et al., 2014). Multiple studies have collectively shown that dynamic loading downregulates catabolic pathways triggered from either injury or cytokine-induced trauma both *in vitro*, *ex-vitro* porcine model and in explanted tissue (Iseki et al., 2019; Hazbun et al., 2021; Engstrom et al., 2022). However, the effectiveness of dynamic loading depends on the amplitude of strain applied. In a pioneering study, Li et al. found that moderate dynamic compression (10%–20%) of bovine cartilage reduced apoptosis and prevented the loss of sGAG compared to non-compressed explants. In contrast, higher amplitudes (30%) resulted in cell apoptosis. It was concluded that lower amplitude compression not only inhibited catabolic responses but also activated anabolic pathways through increased mRNA expression of aggrecan and type II collagen (Li et al., 2013). Interestingly, a study done by Castro-Viñuelas et al. (2024) compared mechanoresponsiveness in OA cartilage to healthy cartilage. This study investigates the role of Wnt signaling in the mechanoresponsiveness of healthy and osteoarthritic human cartilage using *ex-vivo* cartilage explants from human hips (Castro-Vinuelas et al., 2024). According to Cheng et al. (2022), Wnt signaling has been implicated in the pathogenesis of OA. Results indicated that physiological loading maintains anabolic gene expression in non-OA cartilage and that Wnt activation negatively affects chondrocyte mechanoresponsiveness (Cheng et al., 2022). The results from this study indicate that dynamic compressive loading helps maintain cartilage homeostasis even in the presence of OA, but early targeted activation of canonical Wnt signaling impairs the mechanoresponse of chondrocytes.

Dynamic loading has also been shown to affect the viscoelastic properties of AC, a tissue containing both a water liquid phase and collagen/proteoglycan solid phase. As the tissue is loaded hydrostatic pressure increases causing the liquid phase to flow out of the solid phase, creating a frictional drag on the ECM. Healthy AC exhibits biphasic viscoelastic behavior, initially being elastic and allowing fluid movement but becoming viscous over time, resisting the force that squeezes out the interstitial fluid (Sophia Fox et al., 2009). This viscoelasticity results in a conformational change in the shape of the AC requiring recovery time for return to its original configuration. A recent study compared static and dynamic *in situ* loading to evaluate the effect of different loading patterns on AC. T1ρ functional Magnetic Resonance Imaging (MRI) was utilized to quantitatively visualize changes in AC composition and structure. Under these conditions, dynamic loading produced more pronounced structural changes compared to static loading, possibly due to water reabsorption during brief periods of recovery between loading episodes (Truhn et al., 2021). These results are consistent with preclinical studies indicating that dynamic loading leads to a shorter post-loading recovery period, allowing a faster return to full AC thickness and significantly higher minimum cartilage thickness when compared to static loading (Kaab et al., 1998). However, studies incorporating exercise and physical activity are needed to further understand optimal loading patterns post-injury.

It is worth noting that there is an opposite hypothesis that joint distraction, leading to unloading of joints, could ameliorate the clinical symptoms of PTOA and repair the cartilage tissues of the knee joint of the cartilage (Peng et al., 2023; Jansen and Mastbergen, 2022). Notably, Teunissen et al. conducted a study in a canine PTOA model, elucidating that following knee joint distraction (KJD) treatment protocols, there was a restoration in proteoglycan and collagen type II content, accompanied by an increase in proteoglycan synthesis during a 10-week follow-up period (Teunissen et al., 2023). This is likely due to the augmentation observed in the size and density of synovial fluid-derived mesenchymal stem/stromal cell (SF-MSCs) colonies during joint unloading through KJD. Additionally, this unloading triggers a transcriptional shift in SF-MSCs, manifesting as an increase in the expression of the essential cartilage core protein, aggrecan, and a concomitant decrease in the pro-inflammatory chemokine CCL2 (MCP1) (Sanjurjo-Rodriguez et al., 2020). These findings collectively suggest that the temporary unloading induced by KJD induces favorable transcriptional changes in SF-MSCs, potentially facilitating advancements in cartilage regeneration and repair. Overall, KJD studies suggest that temporary joint distraction can create a regenerative environment, likely due to reduced joint loading, allowing cartilage to undergo reparative processes. This leads to a competing hypothesis that the observed benefits may be driven by the modulation of SF-MSCs and their transcriptional changes, which support cartilage regeneration and reduced inflammation. A key question remains regarding the long-term outcomes after reintroduction of mechanical loading. Specifically, a recent study by Teunissen et al. showed in a canine model that 10 weeks after KJD treatment an anabolic cytokine profile was was observed within the joint. However, no study has observed whether or not this anabolic mileau persists after joint reloading (Teunissen et al., 2023).

## 5 Running: a potential role for mechanical loading and physical activity in post-traumatic osteoarthritis

Running is a popular form of exercise that is associated with a variety of health benefits. However, there remains ongoing debate regarding the potential impact of running and its role in the development and progression of OA in both normal and previously injured joints (Roi et al., 2015; Lahr, 1996). Importantly, abnormal joint loading is considered to be a primary catalyst for the development of OA, yet moderate amounts of physical activity and perhaps even the correct dose of rehabilitation can have positive effects on AC. Several human studies have examined the relationship between running and the risk of knee OA finding no significant difference in prevalence, rates, or severity when comparing running to control cohorts (Konradsen et al., 1990; Chakravarty et al., 2008). Epidemiological studies have demonstrated that moderate-intensity running does not cause or worsen OA, while high-intensity running may potentially worsen the disease (Shrier, 2004). A limitation of this study is that the authors only define high intensity running as greater than 20 miles a week while moderate intensity is not specifically defined. In the same context, Willick and Hansen reviewed various studies and found no association between low or moderate intensity running and the risk for development of OA. Interestingly, an association between high-intensity running and OA progression was inconclusive (Willick and Hansen, 2010). Few studies have specifically focused on running and its impact on PTOA. It is worth noting, however, that many of these studies did not consider joint alignment in their patients nor did they document any running gait analysis. Varus and valgus lower extremity alignment are risk factors contributing to the development of medial and lateral knee OA, respectively.

The effects of different running intensities on the development of PTOA has been evaluated in preclinical models. A rodent DMM transection model has been used to assess the effects of treadmill running and limb immobilization on knee cartilage degeneration and joint kinematics (Tsai et al., 2019). The running group demonstrated a greater knee varus angle compared to the control group, while the immobilization group exhibited a reduced rate of voluntary running 2 weeks following surgery. Slow or moderate speed treadmill running did not accelerate PTOA, yet very fast speeds were associated with a slight increase in joint space narrowing. These findings suggest a dose-response relationship between mechanical loading and PTOA development and progression, with moderate-intensity running appearing to have a protective effect on the joint while high-intensity running potentially induces PTOA (Tsai et al., 2019). In another study, Zhou et al. investigated the effects of treadmill running on PTOA development using a rodent model (Zhou et al., 2021). LncRNA H19 was used as a marker for PTOA, as previous studies have shown that reduced H19 expression is associated with OA lesions (Wang et al., 2018). Specifically, moderate intensity treadmill running increased the expression of lncRNA H19 and potentially mitigated motion induced PTOA in mice (Zhou et al., 2021). In contrast, high-intensity treadmill running decreased H19 expression in AC, suggesting an increased risk for the development of PTOA. These findings indicate the importance of different mechanical loading patterns and the potential for their optimization to affect the development and progression of PTOA. Implications for optimal rehabilitation through mechanical loading, including activities such as running and jumping, following injury to optimize AC health is therefore an important perhaps underrecognized therapeutic regimen.

Compared to preclinical studies, there has been less investigation of the effects of running intensity on the development of PTOA in humans. A recent systematic review and meta-analysis synthesized existing studies on the effects of running on cartilage morphology and composition in the tibiofemoral and patellofemoral joints in patients without known trauma. The study acknowledges the ongoing debate about the effects of running on knee joint health, with some studies suggesting a contribution to OA development, while others indicate positive effects such as cartilage regeneration and reduced inflammation. It was concluded that running affects cartilage morphology and composition and is regulated by factors including frequency, intensity, individual age and health, and joint location (Coburn et al., 2023). Generally, running is more associated with cartilage changes in the tibiofemoral joint, while effects on the patellofemoral joint are less clear.

Overall, the relationship between running and knee joint health is complex and multifaceted, with both positive and negative effects observed. However, running serves as a valuable clinical example for understanding the importance of joint loading. A dose-response relationship is consistent across studies, with high-volume running potentially increasing tibiofemoral joint cartilage degeneration. On the other hand, moderate intensity running shows promise in mitigating or even preventing PTOA, highlighting the concept of a U-shaped response where extremes of joint loading/unloading are detrimental and that an optimal loading pattern may be beneficial. Further research is needed to investigate the optimal loading strain and frequency following traumatic joint injuries, such as ACL and meniscal tears, in the context of PTOA development. These studies could advance the field by establishing the relationship between dose of loading (magnitude, duration, frequency) and AC thickness, glycosaminoglycan depletion, and the presence of inflammatory markers.

In terms of the utilization of running for the supplemental management of PTOA, the research is limited. Studies suggest that it is helpful to consider both the factors that contribute to its development after a joint injury and the strategies that have proven effective for preventing injuries and optimizing function in individuals with PTOA (Whittaker and Roos, 2019). Injury prevention programs that involve running exercises, active stretching, controlled partner interactions, planting and cutting movements, and conditioning workouts that focus on strength, agility, and balance, combined with educational elements that stress proper movement patterns during landing and promote fair play, have been shown to significantly lower the incidence of sports-related lower limb injuries.

## 6 Discussion

Both preclinical and clinical studies have shed light on the potential role of orthobiologic therapies in PTOA. Whether genetic variants, sex, and age affect an individual’s response to treatment remains unknown. While there is little clinical data on MSC efficacy in PTOA, animal studies have shown promising results. Trauma-induced OA animal models utilizing various types of MSCs have found improvement in cartilage structure and reduction in PTOA progression. That the anti-inflammatory properties of MSCs may contribute to their therapeutic effects in slowing the progression of OA post-surgically induced trauma. Human studies exploring PRP therapies have reported improvements in quality of life, knee functionality, and reduced pain scores for trauma-related knee osteoarthritis (KOA) patients.

The pro-regenerative capabilities of orthobiologics have been observed for the most part in tightly controlled animal models. Despite the increasing use of MSCs and PRP in clinical practice, the outcomes between animal models and human subjects remain somewhat different. In animal models, regulation of loading patterns post-injection can be carefully controlled, in contrast to the more straightforward approach of injecting cells directly into human knees and lack of monitoring of joint loading. The influence of mechanical loading on chondrocyte behavior and its role in PTOA pathogenesis has been well documented (Sanchez-Adams et al., 2014; Iseki et al., 2019; Hazbun et al., 2021; Engstrom et al., 2022; Truhn et al., 2021; Tsai et al., 2019; Zhao et al., 2021; Davis et al., 2021; Kotelsky et al., 2022; Wu et al., 2023). Within a healthy joint, optimal loading supports chondrocyte survival and matrix synthesis, while excessive loading can lead to cell death and degeneration. Moreover, dynamic loading has demonstrated the most potential for maintaining optimal AC function. Investigations into running and PTOA reveal distinct effects based on different running intensities. High-intensity running negatively impacts knee cartilage health, while low-intensity running also detrimentally affects cartilage health. However, moderate intensity running holds promise in potentially slowing down or even reversing PTOA progression. Understanding the appropriate signaling pathways for these different loading conditions is crucial for advancing our understanding of PTOA and recognizing the pivotal role of mechanical loading in effective treatments.

Optimizing PTOA pathways of treatment includes understanding the role of mechanical loading following injection of orthobiologics. In terms of injectable orthobiologics, a study conducted in 2022 demonstrated the effectiveness of mechanical loading combined with intra-articular injection of adipose-derived MSC in an OA mice model induced by ACL tear (Zhang et al., 2022). The study utilized loading at 1.0 N for 6 min at 5 Hz over a period of 2 weeks. Results indicated that loading enhanced cellular migration, reduced subchondral bone loss, and mitigated AC loss. These findings are consistent with prior research exploring the impact of mechanostimulation on the cellular biology of orthobiologics, particularly MSCs. Dynamic loading, in particular, proves beneficial for MSC chondrogenesis by activating pathways like TGF-β through ALK 5/SMAD 2/3 signaling (Kisiday et al., 2009; Mauck et al., 2007), enhancing ECM components such as aggrecan and collagen type II (Mackay et al., 1998; Huang et al., 2005; Huang et al., 2004). These components, reduced in PTOA progression, are crucial for cartilage health. While highly elevated TGF-β levels may induce hypertrophic maturation in MSCs, limiting clinical utility (Hellingman et al., 2010), dynamic loading, when coupled with TGF-β exposure, reduces hypertrophic markers and limits matrix mineralization in MSCs (Steward et al., 2012; Luo et al., 2015; Ohno et al., 2005). Bioreactor experiments indicate that dynamic loading facilitates MSC mobilization against gravity, aiding their movement from lower to upper compartments (Gamez et al., 2020). Mechanical loading not only influences biochemical pathways and cell migration but also alters the biophysics of individual cells. Normally, chondrocytes exhibit higher viscoelasticity than MSCs, but mechanical stimulation causes MSCs’ viscoelastic properties to resemble those of chondrocytes during MSC chondrogenic differentiation (Fahy et al., 2018; Xie et al., 2019). In addition to this, considering the time course of PTOA, further research is needed to determine the most favorable timing to apply physical therapy/running and/or orthobiologic treatments in order to halt the progression of PTOA. There is currently a paucity of information on this time course. However, such research is difficult to perform as the time course of PTOA development is highly variable and can take as short as 6 months or up to 10–20 years to develop. Future research should also focus on how factors such as age at injury, injury mechanism, and individual biologic factors impact the time course of PTOA development (Kuyinu et al., 2016).

**FIGURE 2:**
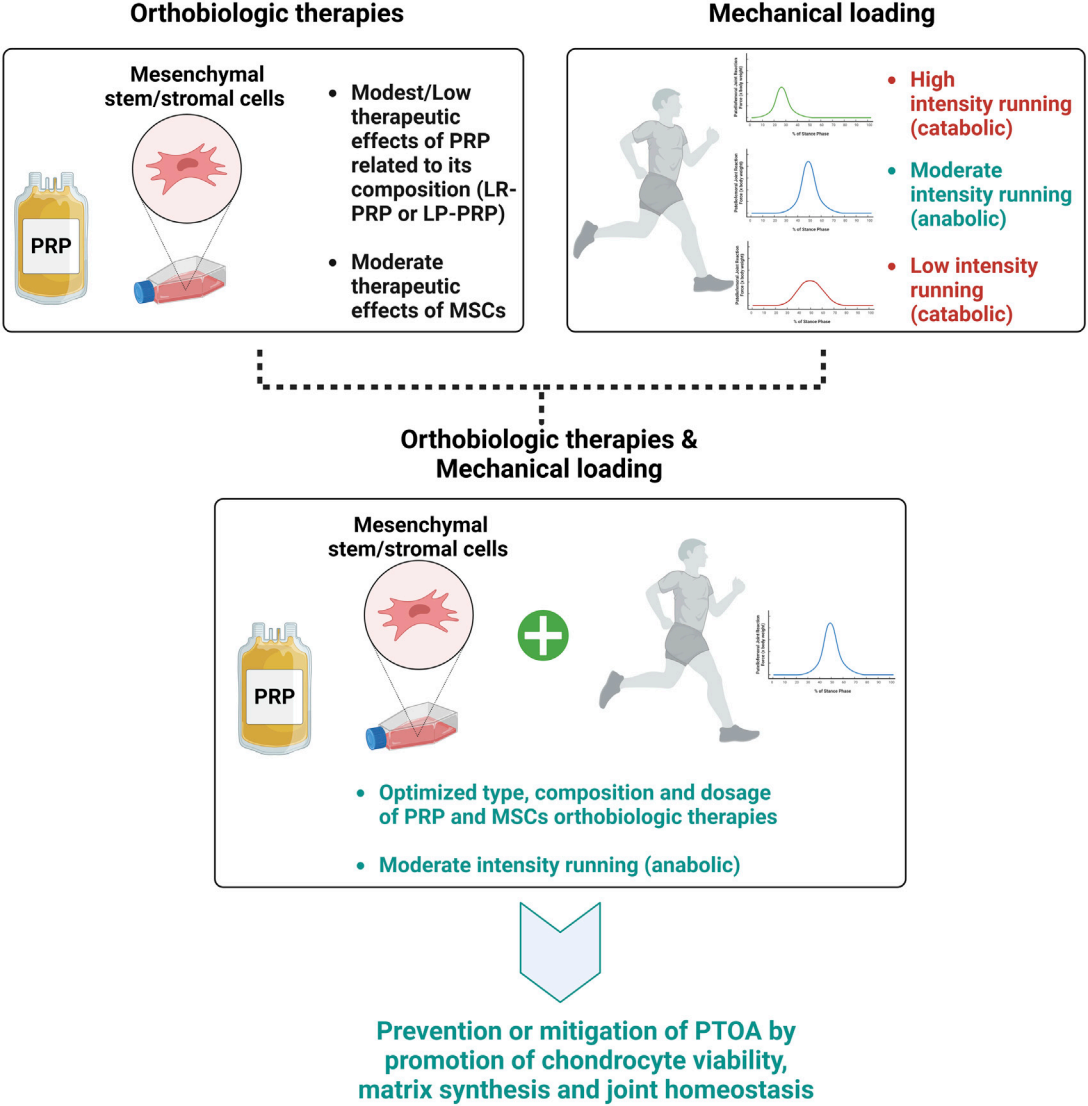
Effects of type, composition, and dosage of orthobiologic therapies and mechanical loading intensity on PTOA development.

Overall, we acknowledge the diverse patient cohorts included in clinical studies, representing a wide range of ages and background comorbidities. It is crucial to recognize the potential influence of these factors on treatment outcomes, and we highlight the importance of understanding patient heterogeneity in interpreting the efficacy of the therapies. However, by combining targeted orthobiologic interventions with mechanical stimulation, there are some encouraging results to date suggesting enhanced treatment outcomes and ideally potentially preventing PTOA development, as illustrated in Figure 2.

## 7 Conclusion

Mechanical loading as a possible strategy in concert with biologic therapies to mitigate PTOA may be a promising addition to our treatment regimen. Mechanical forces influence cellular behaviors and play a significant role in PTOA pathogenesis. Further research into the mechanotransduction pathways involved, as well as optimal loading conditions incorporating physical activity and exercise through strategies such as running, offer promise in pursuit of effective treatment strategies for PTOA. By harnessing the regenerative potential of orthobiologics and optimizing the mechanical environment of the joint, clinicians and scientists can work to improve patient outcomes and contribute to the advancement of PTOA management strategies.
